# Epidemiological and genotypic assessment of cystic echinococcosis in ruminant populations of Northern Punjab, Pakistan: a neglected zoonotic disease

**DOI:** 10.1007/s00436-025-08451-x

**Published:** 2025-01-16

**Authors:** Sakandar Khan, Jo Cable, Numair Masud, Frank Hailer, Muhammad Younus, Naveed Hussain, Muhammad Asif Idrees, Muhammad Imran Rashid, Haroon Akbar

**Affiliations:** 1 Department of Parasitology, University of Veterinary and Animal Sciences, Lahore, Pakistan; 2 Organisms and Environment, School of Biosciences, Cardiff University, Cardiff, UK; 3 Department of Pathobiology, College of Veterinary and Animal Sciences, Narowal, Sub-Campus, UVAS, Lahore, Pakistan; 4 Department of Veterinary Surgery, University of Veterinary and Animal Sciences, Lahore, Pakistan

**Keywords:** *Echinococcus granulosus*, Cystic echinococcosis, Neglected tropical disease, Zoonoses

## Abstract

*Echinococcus* *granulosus* is a zoonotic dog parasite, which causes cystic echinococcosis in humans and animals, adversely affecting health and food production. The resultant socio-economic impacts in lower–middle-income countries and the paucity of epidemiology data prompted the World Health Organization to classify cystic echinococcosis as a neglected tropical disease. Between November 2019 and December 2020, the prevalence of cystic echinococcosis was assessed from 3600 post-slaughter livestock (1142 sheep, 1258 goats and 1200 buffalo) from three districts, Narowal, Sheikhupura and Sialkot, in Northern Punjab, Pakistan. The collected hydatid cysts were subjected to microscopy and PCR followed by Sanger sequencing and phylogenetic analysis. A questionnaire targeted 3600 animal owners to assess disease risk factors. Of the 28 cyst samples sequenced for mtDNA COI, the species was confirmed as *Echinococcus granulosus* and phylogenetic analysis revealed the presence of both G1 and G3 lineages. Overall, we noted a regional difference in prevalence, where infections were significantly higher in Sheikhupura (9.8%) than Sialkot (6.1%) or Narowal (5.9%) districts. Prevalence was higher in buffalo (9.3%) than sheep (7%) or goats (5.5%), significantly higher (9.3%) in older (> 4 years) compared to younger (< 4 years) animals (5.7%) and higher in females (9.1%) compared to males (3.8%). In sheep and buffalo, more cysts were found in the liver (77.7% and 68.7%, respectively), whereas the lungs were more commonly infected in goats (67.1%). Epidemiological data revealed that combining dog deworming with effective disposal of viscera is needed for a significant reduction in infection prevalence. Similarly, animal deworming was only associated with significantly reduced infections when the animals were not kept near an abattoir, where dogs would normally be present and feeding on discarded viscera. This study adds to growing evidence that simple public health measures and awareness are needed to reduce the prevalence of cystic echinococcosis infection.

**Supplementary Information:**

The online version contains supplementary material available at 10.1007/s00436-025-08451-x.

## Introduction

Cystic echinococcosis is a disease of public and animal health concern that is caused by *Echinococcus granulosus* (see McManus et al. 2003). The definitive hosts, dogs, become infected following ingestion of hydatid cyst contaminated organs (Bourée 2001). Intermediate hosts (such as cattle, buffalo, sheep and goats; Lawson and Gemmell 1983) acquire the infection through ingestion of *Echinococcus* eggs that are dispersed from dog faeces (Bourée 2001; Nakao et al. 2010). The disease manifests as fluid-filled cysts within vital organs, mostly the liver and lungs but also the brain, spleen and kidney, causing severe morbidity and even mortality (Battelli 2009). Deemed a neglected tropical zoonosis by the World Health Organization (Beigh et al. 2017), cystic echinococcosis occurs on every continent except Antarctica (WHO 2021).

Annual costs associated with cystic echinococcosis are reported to be US$ 3 billion and yet the true burden of the disease is poorly underestimated in many countries (Budke et al. 2006). In China and Central Asia, more than 20 million people are at risk from cystic echinococcosis (Craig et al. 2007). Where cattle herding is common, the disease is prevalent (Bekele and Butako 2011), ranging from 53.9% in China (Fan et al. 2022), 22% in Ethiopia (Shumuye et al. 2021) to 13.9% in Iran (Vaisi-Raygani et al. 2021) and 12% in India (Grakh et al. 2020). Cystic echinococcosis is also considered prevalent in Pakistan with limited studies revealing prevalence ranging from 2.4 to 35% in different host species across the four administrative provinces of Sindh, Baluchistan, Punjab and Khyber Pakhtunkhwa (Ali et al. 2015; Khan et al. 2022, 2023a, 2023b, 2021b; Mehmood et al. 2020a, 2022; Mustafa et al. 2015; Tasawar et al. 2014). In Sindh and Baluchistan, cystic echinococcosis data is mostly based on human samples (Ullah et al. 2022), with almost no data on livestock infections, just one study in Sindh showing 13.5% prevalence in buffalo populations (Ehsan et al. 2017). More is known about cystic echinococcosis in livestock from Khyber Pakhtunkhwa and Punjab with prevalence rates varying between 2.4 and 17.4% (Haleem et al. 2018; Mustafa et al. 2015). The estimated annual economic loss in Pakistan due to cystic echinococcosis in livestock, mostly through wasted organs (liver and lungs), is > 26.5 million Pakistani rupees (Mustafa et al. 2015). This cost in a country that already faces severe economic hardship, linked to unprecedented inflation (The World Bank, 2023), requires urgent management to minimise socio-economic impacts.

According to current taxonomy (reviewed in Vuitton et al. 2020), *Echinococcus granulosus *sensu lato* (s.l.)* encompasses eight main genotypes (G1, G3–8, G10), with previously described genotypes G2 and G9 currently being designated as microvariants of G3 and G7, respectively. The G1 genotype is most prevalent worldwide and commonly linked to cystic echinococcosis in both humans and livestock (Romig et al. 2015). Global genetic similarity of the G1 genotype suggests that there have been recurrent expansions of this genotype throughout the animal trade (Kinkar et al. 2018a). Higher prevalence of G3 infections though has been observed in Pakistan and India (Alvi et al. 2020; Mehmood et al. 2020b; Muqaddas et al. 2020; Sharma et al. 2013). G3 is predominantly prevalent in buffalo (Mehmood et al. 2020b), but also found in sheep (Kinkar et al. 2018b), goats (Mehmood et al. 2020b), cattle (Alvi et al. 2020; Mehmood et al. 2020b; Kinkar et al. 2018b), camels (Kinkar et al. 2018b; Sharbatkhori et al. 2011), pigs (Mehmood et al. 2021; Pednekar et al. 2009), wild boar (Laurimäe et al. 2019) and humans (Kinkar et al. 2018b; Marinova et al. 2017; Muqaddas et al. 2020), indicating a wide host spectrum and possible expansion of this genotype. In addition, *E. felidis* (formerly ‘lion strain’) isolated from South Africa has been identified as an independent taxon (Huttner et al. 2008).

While there is some data on genetic variants of *Echinococcus granulosus* identified within livestock from the Khyber Pakhtunkhwa and southern and central regions of Punjab Province of Pakistan (see Table 1), there remain gaps in assessment of genetic variants in Northern Punjab in addition to a lack of understanding of epidemiological factors that can be targeted for implementing effective prevention strategies. Given the human toll of this disease, its zoonotic nature necessitates an increased focus on veterinary data. The current study provides an updated assessment of the genotype and prevalence of *E. granulosus* in livestock of Northern Punjab, Pakistan. Specifically, we assessed the parasite genotypes in buffalo, goat and sheep populations of three districts (Narowal, Sheikhupura and Sialkot) that have remained unassessed for *E. granulosus (s.l.)*, and based on questionnaire data, we investigated risk factors associated with this disease.

**Table 1:** Genotypes of *Echinococcus granulosus s.l.* detected from livestock in different regions of Khyber Pakhtunkhwa (KPK) and Punjab provinces of Pakistan

Region	District	Host	Genotypes	References
Punjab	Narowal	Goat	G3	Current study
		Sheep	G1 & G3	
		Buffalo	G1 & G3	
Punjab	Sheikhupura	Goat	G3	Current study
		Sheep	G1 & G3	
		Buffalo	G1 & G3	
Punjab	Sialkot	Goat	G3	Current study
		Sheep	G1 & G3	
		Buffalo	G1 & G3	
Punjab	Lahore	Sheep, goat, camel, cattle	G1	Latif et al. 2010
Punjab	Lahore	Buffalo, sheep, goat	G3	Latif et al. 2010
Punjab	Multan	Cattle	G1 & G3	Mehmood et al. 2022
Punjab	Sialkot	Cattle	G1 & G3	Alvi et al. 2023
Punjab	Sargodha	Goat, sheep, cattle	G1 & G3	Mehmood et al. 2020b
KPK	Peshawar	Human	G1-G3 & G6	Ali et al. 2015

## Materials and methods

### Sample collection

**Fig. 1:**
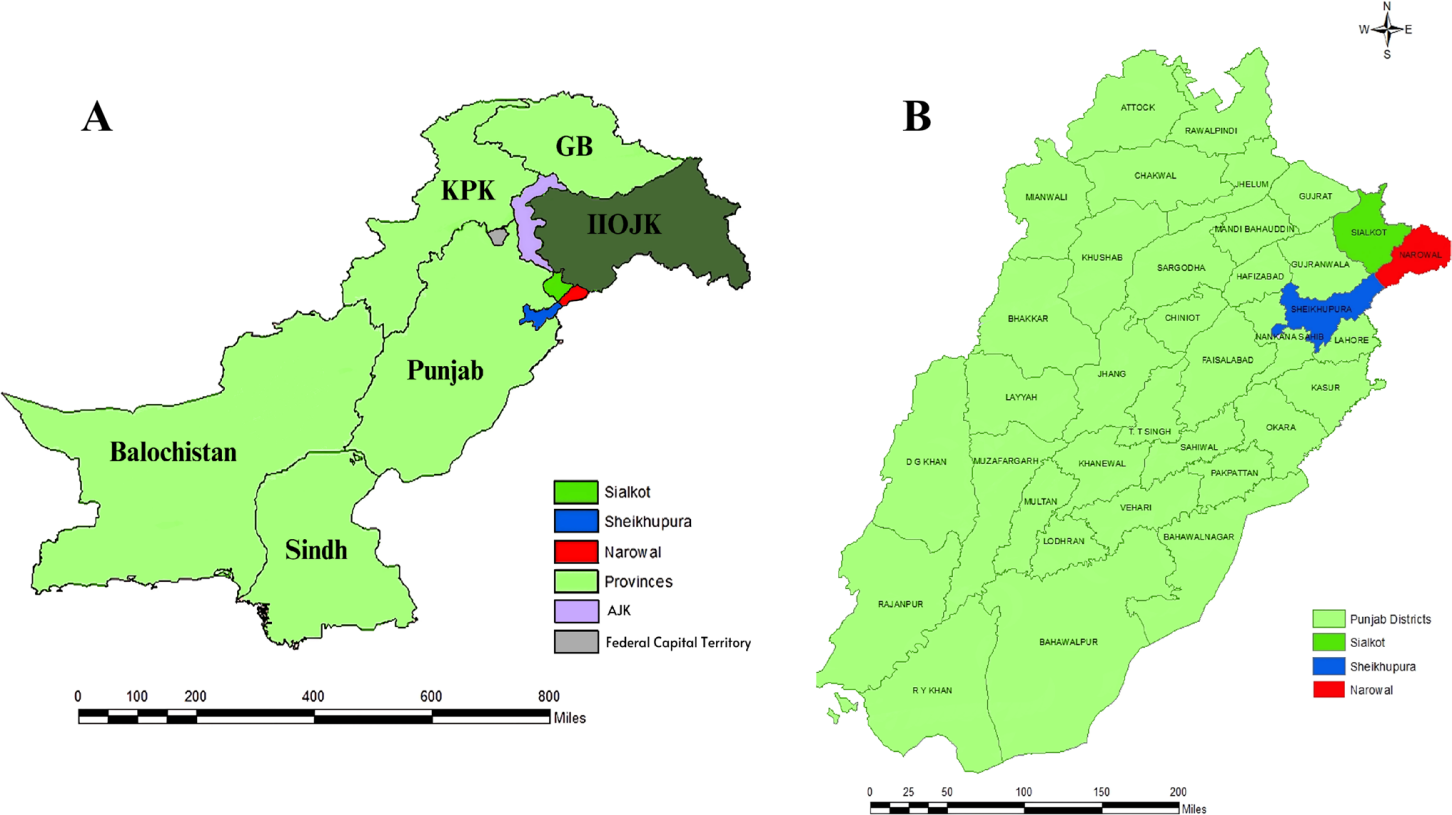
Pakistan sampling districts in the current study. **A** Provinces and **B** districts within the province of Punjab. KPK, Khyber Pakhtunkhwa; GB, Gilgit-Baltistan; and IIOJK, Indian Illegally Occupied Jammu and Kashmir

Between November 2019 and December 2020, parasite (hydatid cyst) samples from 3600 slaughtered animals were collected in three districts, Narowal, Sheikhupura and Sialkot, of Northern Punjab, Pakistan (Fig. 1). From the main slaughterhouse in each district, ca. 400 animals of each species (1142 sheep, 1258 goat and 1200 buffalo, a total of 3600) were grossly examined for the presence of hydatid cysts. We recorded the host species, age and sex, the presence of cysts (yes or no) and anatomical location of cysts. We collected one cyst per organ per animal. Although some organs (liver and lungs) had multiple cysts, these were not collected or counted.

The liver, lungs, spleen, heart, and kidneys with cysts were placed in containers with 70% ethanol and transported to the Department of Parasitology, UVAS, Lahore, for further examination. Each hydatid cyst was aspirated aseptically, and wet mounts examined by light Olympus microscope (CX21FS1) at × 100 and × 400 magnification. The cysts were recorded as fertile or sterile if the protoscoleces were or were not detected, respectively.

### Molecular identification and phylogenetic analysis

Genomic DNA was extracted from hydatid cysts using WizPrep™ gDNA Tissue kits (Wizbiosolutions, South Korea, REF # W71060-300). The extracted DNA was quantified using a NanoDrop 1000 spectrophotometer (Thermo Scientific, USA). Next, PCR was performed in a SimpliAmp thermal cycler (Applied Biosystems), using primers JB3 (5′-TTTTTTGGGCATCCTGAGGTTTAT-3′) and JB4.5 (5′-TAAAGAAAGAACATAATGAAAATG-3′) (Bowles et al. 1992), which target a 446 bp fragment (incl. primers) of the mtDNA CO1 gene. This primer pair is widely used to amplify genotypes of *E. granulosus (s.l.)* (see Moks et al. 2008).

Reactions were conducted in total reaction volumes of 10 µL, containing 1.5 µL genomic cyst DNA, 5 µL of 2 × Qiagen Multiplex PCR mix (Qiagen, Hilden, Germany), 0.4 µL of each forward and reverse primer solution (10 µM) and 2.7 µL nuclease free water (VWR). The PCR conditions were initial denaturation at 95 °C for 15 min (1 cycle), followed by denaturation at 94 °C for 45 s, and annealing in a touchdown approach at 60–50 °C for 45 s (with the annealing temperature decreasing by 0.5 °C each cycle), and extension at 72 °C for 1 min for 20 cycles. This was followed by another 20 cycles of denaturation at 94 °C for 45 s, annealing at 50 °C for 45 s and extension at 72 °C for 1 min. The PCR concluded with a final extension step at 72 °C for 10 min. The PCR products were visualized by running them on 1% agarose gels containing SYBR safe dye (Invitrogen USA) and observing them under UV light using a GelDoc-It™ system (P/N 97–0266-02, Upland, CA, USA).

PCR products were randomly selected for Sanger sequencing at a commercial sequencing facility (Eurofins Genomics services, Germany), following a 1:5 dilution with water (3 µL PCR product, 12 µL water and 2 µL forward primer). The resulting chromatograms were checked for quality, trimming low-quality bases at the ends, and coding any ambiguous characters with IUPAC codes. Out of 38 samples selected for Sanger sequencing, 28 were of sufficient quality for further analysis. After quality trimming and inclusion of G1-G10 reference sequences from across the *Echinococcus* phylogeny by Zhao et al. (2022) from GenBank, we obtained an alignment of 371 bp, excluding primers. These sequences have been deposited in GenBank (Accession Numbers PQ537127-PQ537154). *Versteria mustelae* (AB732957) was used as an outgroup for the genus *Echinococcus* (see Knapp et al. 2011). We aligned the downloaded CO1 sequences with our newly obtained sequences using the MUSCLE plugin within Geneious Prime (version 2022.2.2; www.geneious.com).

A maximum likelihood phylogenetic tree was constructed using IQ-tree webserver (version 2.2.0) (Trifinopoulos et al. 2016). The appropriate substitution model for our alignment was determined by the built-in model finder function (yielding the TN + F + G4) (Kalyaanamoorthy et al. 2017), and statistical support for branches was determined from 1000 ultrafast bootstrap replicates (Hoang et al. 2018). For construction of median-joining phylogenetic networks (Bandelt et al. 1999), the aligned sequences were analyzed in PopART (http://popart.otago.ac.nz), setting ε (reticulation factor) to zero.

### Questionnaire for risk factor analysis

A questionnaire was developed for risk factor analysis that included 18 simple closed-ended questions regarding owner and animal details. During face-to-face discussions in local dialect at the slaughterhouses, 3600 farmers were surveyed, each representing a distinct farm from where the animals originated. They were asked about potential risk factors such as the district of their farm, the habitat of the farm (peri-urban, urban, or rural), ownership of dogs, practices regarding dog deworming and home slaughtering, methods of offal disposal (burial, leaving in the open, or unknown), the practice of feeding viscera to dogs, disposal methods of dog faeces, distance from the abattoir, deworming routines for livestock and the feeding patterns of animals (confined, mixed or grazing) (Khan et al. 2023b).

### Statistical analyses

All statistical analyses were conducted using RStudio (Version 2023.09.0 + 463). All epidemiological data was analyzed using generalised linear models (GLMs) with binomial error families as the response variable was binary (presence/absence of infection and fertility of cysts-yes/no data; see Table 2 for model details).

**Table 2:** Generalized linear model (GLM) analyses were conducted to study the prevalence of cystic echinococcosis in slaughtered sheep, goats, and buffaloes. Risk factors were assessed using data from a sociodemographic survey conducted among farmers concurrently with the sample collection

Risk factors	Response	No. of positive/total (%)	SE	*Z* value	Odds ratio	*P* value
**Models 1:** The GLM with loglink function and binomial error families explored the risk factors associated with location, age, sex, and species. Narowal and buffalo are reference categories for districts and species respectively						
Districts	Sheikhupura	118/1200 (9.8)	0.15	2.82	1.56	0.004
	Sialkot	74/1200 (6.1)	0.17	− 0.47	0.91	0.63
	Narowal	71/1200 (5.9)				
Age (years)	< 4	71/1887 (3.7)	0.15	7.63	3.22	0.001
	> 4	192/1713 (11.2)				
Sex	Males	49/1267 (3.8)	0.16	− 3.27	0.57	0.001
	Females	214/2333 (9.1)				
Species	Buffalo	112/1200 (9.3)				
	Sheep	81/1142 (7.0)	0.16	− 3.25	0.59	> 0.05
	Goat	70/1258 (5.5)	0.16	− 2.19	0.48	0.02
**Model 2**: Risk factors related to habitat, feeding, deworming, slaughtering, offal disposal, and distance from abattoir were analyzed using generalized linear model with loglink function and binomial error families. The peri-urban, confined, buried and 10–20 km were reference categories for habitat, animal feeding, disposal of offal and distance from abattoir, respectively						
Habitat	Peri-urban	10/621 (1.6)				
	Rural	221/1902 (11.6)	0.35	7.17	12.17	0.001
	Urban	32/1077 (2.9)	0.38	2.18	2.30	0.02
Animal feeding	Confined	25/667 (3.7)				
	Mixed	168/2629 (6.39)	0.25	1.48	1.46	0.13
	Grazing	70/304 (23.02)	0.27	6.65	6.31	0.001
Deworming of animals	Yes	123/1505 (8.1)	0.18	−0.36	0.93	0.71
	No	140/2095 (6.6)				
Home slaughtering	Yes	251/3023 (8.3)	0.40	−1.74	0.49	0.08
	No	12/577 (2)				
Disposal of offal	Buried	79/1387 (5.6)				
	Left open	172/1636 (10.51)	0.14	4.31	1.94	0.01
	Unknown	12/577 (1.7)	0.31	−3.30	0.35	0.02
Distance from abattoir	3–5 km	145/1066 (13.6)	0.19	8.34	4.90	0.001
	5–10 km	32/1236 (2.5)	0.24	−2.18	0.59	0.02
	10–20 km	86/1298 (6.6)				
**Model 3**: The risk factors associated with dogs were investigated using GLM with loglink function and binomial error families, with the reference categories being no dog for deworming and feeding viscera to dogs. For the variable of faeces disposal, the reference category was labelled as unknown						
Keeping of dogs	Yes	204/1150(17.7)	0.15	14.19	8.73	0.001
	No	59/2450 (2.4)				
Deworming of dog	No dog	59/2450 (2.4)				
	Yes	39/234 (16.6)	0.21	9.53	8.10	0.001
	No	165/916 (18)	0.15	13.89	8.90	0.001
Feeding dogs with viscera	No dog	59/2450 (2.4)				
	Yes	191/842 (22.6)	0.15	15.94	11.91	0.001
	No	13/308 (4.2)	0.31	1085	1.7	0.063
Disposal of dog faeces	Unknown	59/2450 (2.4)				
	No	151/452 (33.4)	0.16	18.22	20.33	0.001
	Yes	53/698 (7.5)	0.19	6.18	3.32	0.001

The independent variables in the GLMs included regional districts in Northern Punjab, age and sex of animals, species (goat, sheep or buffalo), deworming (yes/no), type of animal feeding (open grazing, confined feeding, or combination of the two), slaughtering of animals at home (yes/no), offal disposal (yes/no) and distance from slaughterhouse. Keeping dogs at home or with goat, sheep or buffalo (yes/no), deworming dogs (yes/no), viscera feeding to dogs (yes/no), and disposal of dog faeces (yes/no) were also included as independent variables. Furthermore, the association between the fertility of cysts and species and anatomical location was also assessed using binomial GLMs.

## Results

### Genotyping of *Echinococcus *(*cytochrome oxidase* 1 gene)

**Fig. 2:**
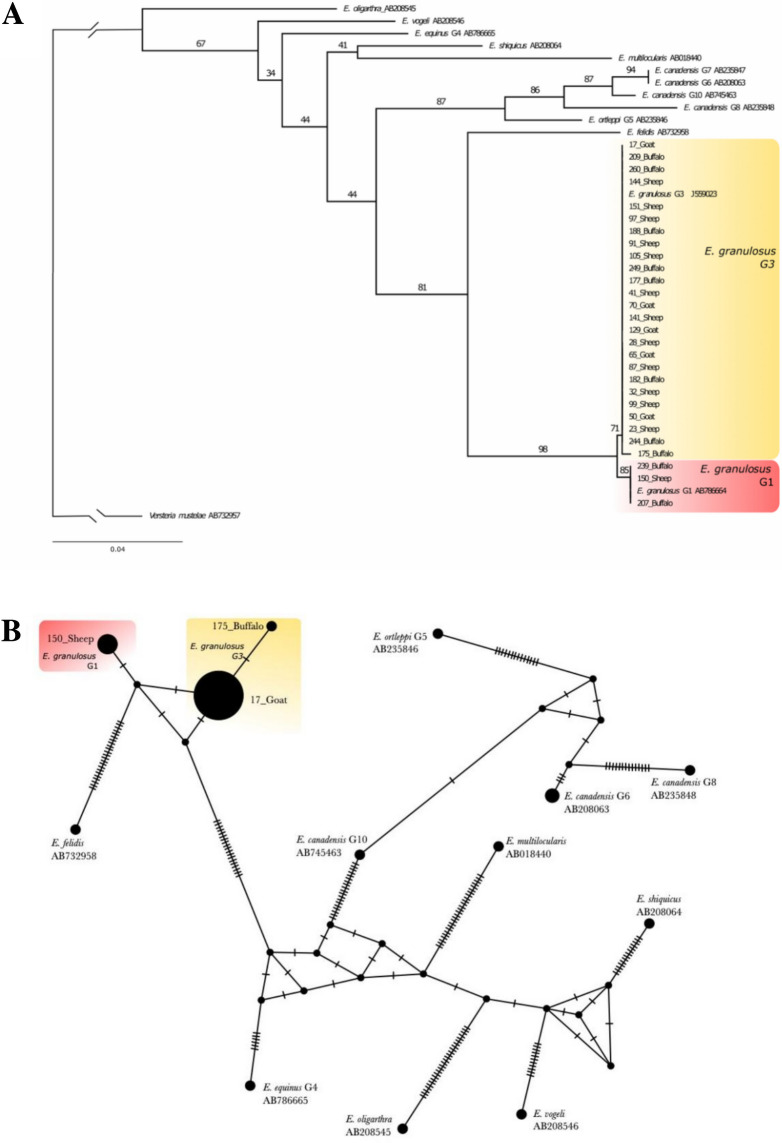
Phylogenetic analysis of a 371 bp alignment of CO1 sequences in *Echinococcus species*, based on sequences from the present study and representative lineages from Zhao et al. (2022). Sequences from GenBank include the lineage name (G1-G10; Zhao et al. 2022) and Genbank accession number. **A** Maximum-likelihood phylogeny from IQ-tree (Trifinopoulos et al. 2016), with *Versteria mustelae* as outgroup. Numbers on branches denote ultrafast bootstrap support values; values within the G1 and G3 lineages that were < 50 have been removed for clarity. **B** Median-Joining network (Bandelt et al. 1999) of *Echinococcus granulosus s.l.* isolates

The presence of *Echinococcus granulosus (s.l.)* was confirmed via COI gene sequencing. Maximum likelihood phylogenetic analysis (Fig. 2A) revealed that three of our sequences (two buffalo and one sheep) grouped with G1 (3/28; 10.7%) with an ultrafast bootstrap support (UF) of 85. The remaining 25 sequences (8 buffalo, 5 goat and 12 sheep) grouped with the G3 genotype (25/28; 89.2%; UF 71). Support for the G1 and G3 lineages clustering together was UF = 98. Network analysis (Fig. 2B) revealed that the main G1 and G3 haplotypes were separated by two fixed substitutions. Within G3, we found two haplotypes, one within a single host (175_buffalo), separated by one mutation from the dominant G3 haplotype.

### Epidemiological data

In Northern Punjab, significantly more cystic echinococcosis prevalence was noted in Sheikhupura (9.8%; *p* = 0.004) compared to Sialkot (6.1%; *p* = 0.63) and Narowal (5.9%) (Table 3). Overall, buffalo had significantly higher prevalence (9.3%) than goats (5.5%; GLM: std. err = 0.38, *z* value = − 2.19, *p* = 0.02), but no significant differences were noted between sheep (7%) and buffalo or goats.

**Table 3:** Individual distribution of *Echinococcus* positive animals based on post-slaughter examination categorized by host species, districts, sex, and age groups

Variable	Response	Buffalo	Sheep	Goat
**Districts**	Narowal	30/400 (7.5%)	24/380 (6.3%)	17/420 (4.0%)
	Sheikhupura	43/400 (10.7%)	36/380 (9.4%)	39/420 (9.2%)
	Sialkot	39/400 (9.7%)	21/382 (5.4%)	14/418 (3.3%)
**Sex**	Male	28/308 (9.0%)	16/513 (3.1%)	5/446 (1.1%)
	Female	84/892 (9.4%)	65/629 (10.3%)	65/812 (8%)
**Age (years)**	< 4	50/791 (6.3%)	12/506 (2.3%)	9/590 (1.5%)
	> 4	62/409 (15.1%)	69/636 (10.8%)	61/668 (9.1%)

Significant interactions were noted between the age and sex of animal in relation to prevalence, where males of all species above 4 years of age had significantly fewer infections compared to females at all ages (GLM: std. err = 0.47, *z* value = − 1.98, *p* = 0.04). Indeed, animal age as a factor was the most significant explanatory variable of disease compared to sex, regional district or species (GLM: std. err = 0.22, *z* value = 5, *p* > 0.001; Table 2) for age of all animal species assessed in this study. No significant association was noted between the fertility of the hydatid cysts recovered via autopsy and specific organs; however, there were more viable fertile hydatid cysts in sheep (GLM: std. err = 0.21, *z* value = 3.21, *p* = 0.001; see Table 4 for hydatid cyst organ distribution) than in goats and buffalo.

**Table 4:** Fertility and sterility of *Echinococcus granulosus* cysts obtained from different organs of slaughtered buffalo, sheep, and goat in the current study

Species	Buffalo (*n* = 1200)			Sheep (*n* = 1142)			Goat (*n* = 1258)		
	Number of fertile, sterile, and calcified cysts (%) per host species								
	Fertile	Sterile	Cal	Fertile	Sterile	Cal	Fertile	Sterile	Cal
**Liver**	46 (41)	27 (24.1)	4 (3.5)	53 (65.4)	10 (12.3)	0	11 (15.7)	3 (4.2)	1 (1.4)
**Lungs**	19 (16.9)	12 (10.7)	4 (3.5)	11 (13.5)	5 (6.1)	1 (1.2)	47 (67.1)	0	0
**Heart**	0	0	0	0	0	0	2 (2.8)	0	1 (1.4)
**Kidney**	0	0	0	0	2 (2.4)	0	1 (1.4)	2 (2.8)	0
**Spleen**	0	0	0	0	0	0	1 (1.4)	1	0
**Total**	112			81			70		

Animals fed via grazing had significantly higher prevalence of infection compared to those fed under confinement or a mixture of confinement and grazing (GLM: std. err = 0.29, *z* value = 5 0.47, *p* < 0.001). Significantly lower infection prevalence was noted in all animals that were both dewormed and at a greater distance from the abattoirs (*p* = 0.04), whereas this association was not found when deworming occurred at a closer distance to the abattoir.

Moreover, no significant association was found between infection prevalence and the practice of disposing of viscera after slaughter (*p* > 0.05). There was a significant association between increased prevalence of infections in all species and the feeding of discarded viscera to dogs (*p* < 0.001). Although deworming of dogs and the effective disposal of viscera independently were not associated with any significant reduction of infection, there was significant interaction between the two variables, indicating that deworming dogs and safe disposal of viscera are needed in combination for a significant reduction in infection (*p* < 0.001).

## Discussion

We confirm the presence of *Echinococcus granulosus* in the Northern Punjab province of Pakistan. Specifically, we observed both G1 (sheep and buffalo) and G3 (goat, sheep and buffalo) genotypes, consistent with previous studies identifying G1 and G3 in South Punjab (Mehmood et al. 2022), the Lahore district of Punjab (Latif et al. 2010) and Khyber Pakhtunkhwa (Khan et al. 2021a). In contrast to lineage frequencies globally, G1 (10.7%) was less common than G3 (89.2%) in our dataset. This distinct distribution of G1 and G3 genotypes could result from geographic isolation linked to the Himalayan Mountain chain, acting as a barrier between Central/East Asia and South Asia, hindering animal movement and trade, but also parasite transmission (Mehmood et al. 2022). This hypothesis though remains speculative due to the lack of high-resolution data in the wider geographic region.

In Northern Punjab, cystic echinococcosis was most common in the district of Sheikhupura than Narowal or Sialkot. This geographical variation in disease occurrence may reflect regional variations in environmental, social and/or economic factors. Temperature and humidity are the key environmental factors known to influence occurrence of this disease (Torgerson and Heath 2003). The current study confirms that deworming of animals and increased distance from slaughterhouses is associated with decreased infection prevalence (Yang et al. 2015). Deworming of dogs alone was not associated with significant reduction in infection cases, but combining effective disposal of viscera with deworming of dogs was associated with reduced incidences of infection. This emphasises the need to combine effective management of *Echinococcus* infections with not only medical interventions but also the implementation of simple hygiene practices (Gemmell et al. 2001), which in rural areas in particular must be linked to educational campaigns (Pinto et al. 2020; Khoshgoftar et al. 2021). Contrary to Azlaf and Dakkak 2006, we did not find that home slaughtering increased the risk of infection. This may be because people are now slaughtering animals within a designated place and correctly disposing of waste and condemned organs.

In all districts, buffalo had a higher infection rate than sheep and goats (current study Table 3; Zhao et al. 2022). Sheep harboured more fertile cysts than buffalo and goats, possibly reflecting adaption of the different genotypes to different host species (Mehmood et al. 2020a). Overall, we identified more non-viable than fertile cysts; it is not clear why this was the case as other studies reported the reverse (Anwar et al. 2000; Latif et al. 2010). In all species, animals older than four years had a higher number of cysts and a greater prevalence compared to younger animals reflecting increased exposure time of older animals to the parasite (Khan et al. 2023a; Mahmood et al. 2022). Higher infection in female than male animals, confirmed in this study, was expected given that (i) females are kept alive for longer for milk production and so are exposed to the parasite for a greater proportion of their lives (Ehsan et al. 2017), whereas males are slaughtered at an early age (Khan et al. 2010), (ii) pregnancy, parturition and lactation might increase disease susceptibility (Khan et al. 2023a) and (iii) variation in livestock management means females are usually managed near human habitation and so are in closer contact with dogs (Haleem et al. 2018). Higher infection in animals primarily fed via grazing was probably linked to greater chance of consuming parasites in contaminated pasture. Greater investment in agriculture infrastructure with ethical confinement of ungulates for targeted feeding would limit exposure to biohazards; however, research into the efficacy of such interventions is needed, and for lower–middle-income countries (LMICs), it is not currently feasible.

For *E. granulosus*, prevalence is heightened by interacting socioeconomic factors, particularly the lack of necessary sanitation infrastructure with improper disposal of human and animal waste (which is consumed by dogs), and lower literacy rate in rural areas tends to lead to higher levels of water and food contamination with *E. granulosus* eggs (Javed and Alkheraije 2023; Khan et al. 2020, 2018; Stephens et al. 2023). For future studies, it would be useful to include a wider array of slaughterhouses in each district (compared to just the main one used for this study). Secondly, as only a small number of goat samples were sequenced, we do not know whether the G1 genotype is present in this species within the studied area. Thirdly, this study targeted only a short sequence of the CO1 gene, so to clearly assess the distribution and origin of different *E. granulosus* genotypes, it would be useful to compare complete mitochondrial genomic sequences (Basuony et al. 2024) or the whole genome (du Plessis et al. 2023).

## Conclusion

Cystic echinococcosis is prevalent in Northern Punjab, Pakistan ranging from 5.6 to 9.6% in livestock from Narowal, Sialkot and Sheikhupura with higher incidence in buffalo, compared to sheep and goats, and in older animals. *E. granulosus* high prevalence in Sheikhupura might be related to higher stocking levels of buffalo compared to other districts but no updated livestock census data is currently available. *E. granulosus* can be effectively managed (deworming of animals, proper disposal of offal and effective management of faecal waste) but often requires improved investment and development of sanitation, infrastructure and increased awareness of prevention strategies, particularly among rural populations.

## Supplementary Information

Below is the link to the electronic supplementary material.

Supplementary file1 (PPTX 69.8 KB)

## Data Availability

All newly obtained DNA sequences have been deposited on Genbank (Accession Numbers PQ537127-PQ537154). The epidemiological data is available from the corresponding author upon request.
