# Supplementary material for: Epidemiological and genotypic assessment of cystic echinococcosis in ruminant populations of Northern Punjab, Pakistan: a neglected zoonotic disease
Source: Parasitol Res. 2025 Jan 16;124(1):7. doi: 10.1007/s00436-025-08451-x (PMC11739240; doi:10.1007/s00436-025-08451-x)
Supplement: Supplementary file 1 — Supplementary file1 (PPTX 69.8 KB) [file 436_2025_8451_MOESM1_ESM.pptx]

## Slide 1
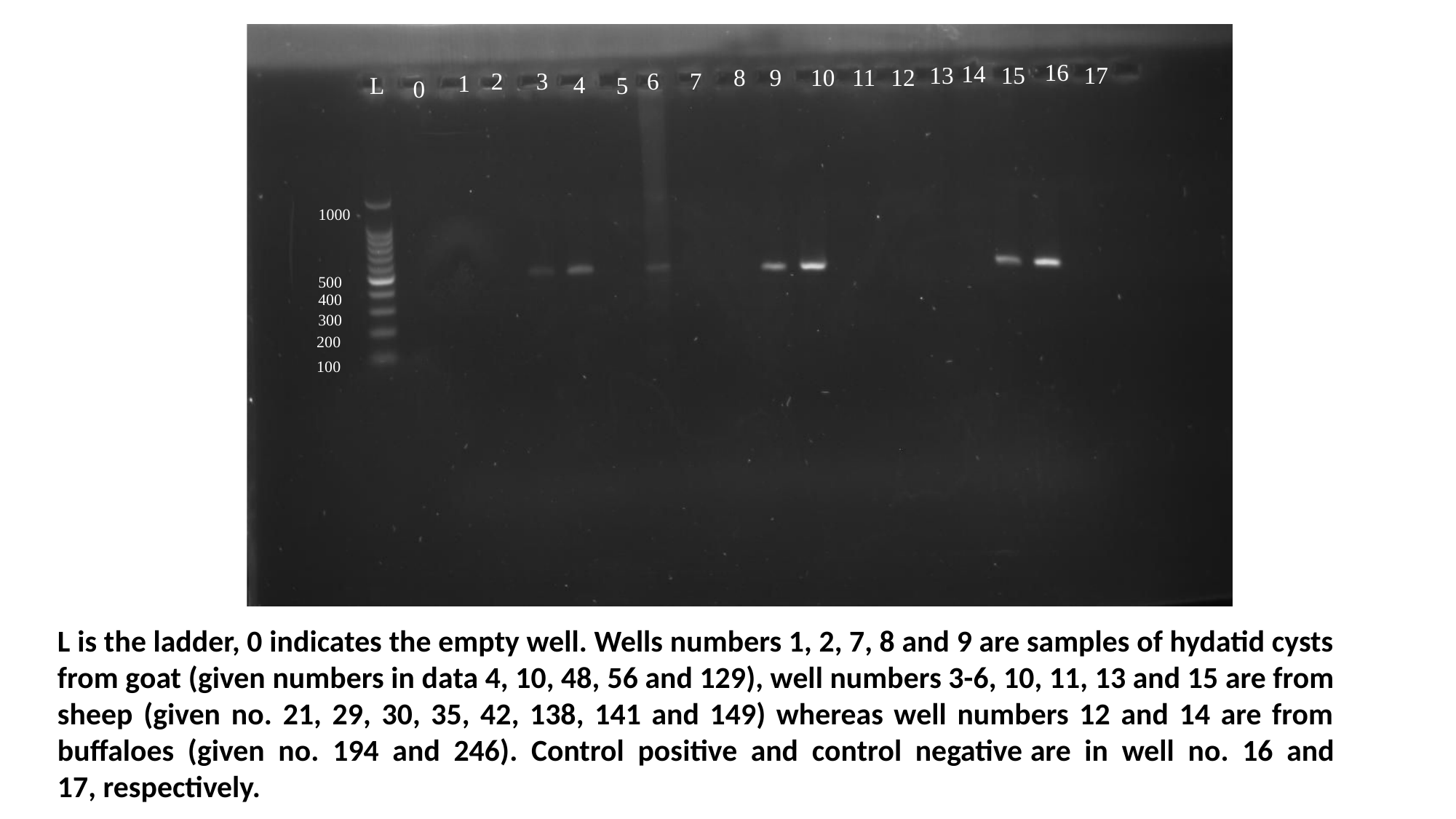

16
14
13
15
17
8
9
10
11
12
2
3
6
7
1
4
L
5
0
1000
500
400
300
200
100
L is the ladder, 0 indicates the empty well. Wells numbers 1, 2, 7, 8 and 9 are samples of hydatid cysts from goat (given numbers in data 4, 10, 48, 56 and 129), well numbers 3-6, 10, 11, 13 and 15 are from sheep (given no. 21, 29, 30, 35, 42, 138, 141 and 149) whereas well numbers 12 and 14 are from buffaloes (given no. 194 and 246). Control positive and control negative are in well no. 16 and 17, respectively.
